# HitWalker: variant prioritization for personalized functional cancer genomics

**DOI:** 10.1093/bioinformatics/btt003

**Published:** 2013-01-09

**Authors:** Daniel Bottomly, Beth Wilmot, Jeffrey W. Tyner, Christopher A. Eide, Marc M. Loriaux, Brian J. Druker, Shannon K. McWeeney

**Affiliations:** ^1^Knight Cancer Institute, ^2^Oregon Clinical and Translational Research Institute, ^3^Department of Cell and Developmental Biology, ^4^Division of Hematology and Medical Oncology, ^5^Howard Hughes Medical Institute and ^6^Department of Anatomic Pathology, Oregon Health and Science University, Portland, OR 97239, USA

## Abstract

**Summary:** Determining the functional relevance of identified sequence variants in cancer is a prerequisite to ultimately matching specific therapies with individual patients. This level of mechanistic understanding requires integration of genomic information with complementary functional analyses to identify oncogenic targets and relies on the development of computational frameworks to aid in the prioritization and visualization of these diverse data types. In response to this, we have developed HitWalker, which prioritizes patient variants relative to their weighted proximity to functional assay results in a protein–protein interaction network. It is highly extensible, allowing incorporation of diverse data types to refine prioritization. In addition to a ranked list of variants, we have also devised a simple shortest path-based approach of visualizing the results in an intuitive manner to provide biological interpretation.

**Availability and implementation:** The program, documentation and example data are available as an R package from www.biodevlab.org/HitWalker.html.

**Contact:** bottomly@ohsu.edu

**Supplementary information:** Supplementary data are available at *Bioinformatics* online.

## 1 INTRODUCTION

The advent of next-generation sequencing technology such as that available from Illumina provides an unprecedented ability to interrogate individual genomes (Metzker, 2010). Accompanying technologies such as the ability to multiplex samples, as well as efficient sequence capture technologies (Ng *et al.*, 2009) further enable targeted regions of the genome to be re-sequenced at a reasonable cost per sample. In this manner, specific genes or whole exomes can be interrogated to identify variants potentially having a deleterious impact on protein coding regions. Efficacy of this approach for clinical research has been shown through the discovery of variants underlying simple Mendelian disorders, as well as more complicated variants/mutations involved in cancer and potentially complex traits (Kiezun *et al.*, 2012). Because many variants are produced for a given sample, mechanistic and population genetic assumptions are often used to reduce the set of variants (Ng *et al.*, 2009). For instance, limiting attention to non-synonymous single-nucleotide variations, as well as using the use of variant databases, such as dbSNP (Sherry *et al.*, 2001) and the 1000 genomes project (The 1000 Genomes Project Consortium, 2010), enables researchers to focus on low-frequency variants that are potentially damaging. However, even after these filters, it is relatively infrequent that a researcher is left with a manageable set of variants for biological validation.

For cancer cells derived from a given patient, functional assays can be performed that allow researchers to determine the relevance of genes to cell viability, such as through the use of targeted siRNA screens (Tyner *et al.*, 2009). These gene targets can be scored using a binary or quantitative encoding that indicates outlier status relative to other samples. Similarly, screens that measure the sensitivity of a patient’s cells to a panel of small molecules can be scored relative to genes using the known gene targets of each compound (Tyner *et al.*, 2013). These functional assays by themselves provide some information to researchers on the identity of the signalling pathways that are required for cancer growth and survival. However, they do not necessarily indicate the mutated gene(s) leading to dysregulation of these signalling pathways, as the true causative variant(s) could be found in any gene with capacity to regulate the pathway.

To prioritize variants detected in our cohort of leukaemia patients relative to our functional assay results, we have devised the R and SQL framework HitWalker using a protein–protein interaction (PPI) network as a backbone. In addition to a ranked list of variants, HitWalker also allows for simple visualization of the relevant subnetwork, as well as overlaying relevant meta-data.

## 2 DESCRIPTION OF SOFTWARE

### 2.1 Variant prioritization

Variants are related to genes implicated from the functional assays through a user-defined PPI network, such as STRING (Szklarczyk *et al.*, 2011), which provides known and hypothetical links between proteins that are weighted by a confidence score. Variants and the functional assay results are mapped to the proteins of the network using pre-supplied meta-data. For example, variants can be mapped to transcripts, which in turn can be mapped to proteins (as well as gene symbols). This hierarchy is managed through the specification of ‘core’ or ‘summary’ IDs to be used in the internal functions.

By default, prioritization is performed using a random walk with restarts algorithm (RWR) (Köhler *et al.*, 2008; Tong *et al.*, 2008). In HitWalker, RWR provides a measure of weighted proximity between a set of proteins associated with functional assay hits and a set of proteins containing variants. Variants are prioritized based on the resulting RWR association score attributed to the protein. See the Supplementary Material for more information. We note that any number of approaches related to our RWR implementation could be easily applied as well, including corrections for degree (Erten *et al.*, 2011).

### 2.2 Visualization

**Fig. 1.**
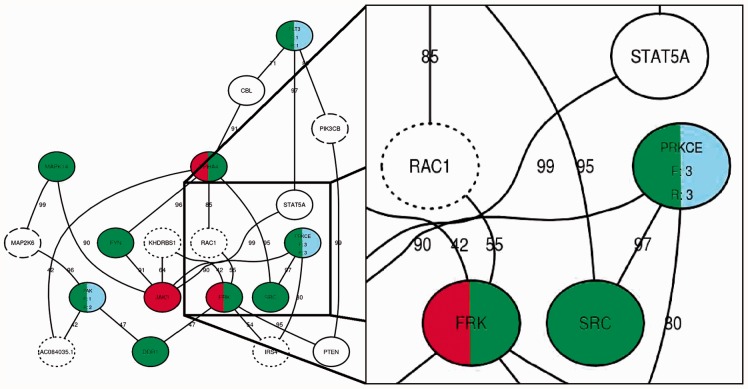
Modified visualization output from HitWalker displaying the top three assay hits (EPHA4, JAK3 and FRK) and variants (FLT3, ZAK and PRKCE) for an acute myeloid leukaemia patient. Note that other hits are pulled out and annotated, as they are on the shortest path. Gene names are provided for each node. For nodes containing variants (blue), frequency information is reported in terms of the patient cohort counts (F), as well as the RWR rank (R). Red and green nodes indicate siRNA and gene target hits, respectively. Dotted borders indicate absence of capture probes for a given gene. Dashed borders indicate functional assay targets whose inhibition did not significantly alter cell viability. Confidence scores for the interactions between the two genes are reported near the lines connecting two given genes

The relevant subnetworks can be visualized by approximating the RWR algorithm using the unweighted shortest paths between the top queries and seeds. Shortest paths connecting the functional hit and variant genes are also displayed for additional context. For the shortest path calculations, the path with the highest overall path score is chosen if multiple equivalent shortest paths exist. The user can define functions mapping meta-data in the database to attributes of the network. For instance, genes can be represented using different shapes and colours. An example of such a figure is shown in Figure 1 and Supplementary Figure S1. In a patient with acute myeloid leukaemia, an S451F amino acid change in the FLT3 gene was the top ranking variant, which has been previously characterized and catalogued in COSMIC (Forbes *et al.*, 2011). Non-synonymous single-nucleotide variants in ZAK and PRKCE were the next highest-ranked variants, and all three genes are candidates based on the observed drug response.

## 3 DISCUSSION

Our software provides a flexible framework for prioritizing variants relative to functional assay outcomes and a PPI network or other association graph using an RWR algorithm. In addition, we include visualization of key subnetwork members to aid in biological interpretation. Prioritized variants can then be followed up using Sanger sequencing, as well as additional experiments to categorize their phenotypic effect. Software developers can easily modify queries and R functions for their in-house databases and functional assays. Although MySQL is our database of choice, any other R/DBI-supported database would work with minor modifications to the R code.

## Supplementary Material

Supplementary Data

## References

[btt003-B1] Erten S (2011). DADA: Degree-Aware Algorithms for Network-Based Disease Gene Prioritization. BioData Mining.

[btt003-B2] Forbes SA (2011). COSMIC: mining complete cancer genomes in the Catalogue of Somatic Mutations in Cancer. Nucleic Acids Res..

[btt003-B3] Kiezun A (2012). Exome sequencing and the genetic basis of complex traits. Nat. Genet..

[btt003-B4] Köhler S (2008). Walking the interactome for prioritization of candidate disease genes. Am. J. Hum. Genet..

[btt003-B5] Metzker ML (2010). Sequencing technologies: the next generation. Nat. Rev. Genet..

[btt003-B6] Ng SB (2009). Targeted capture and massively parallel sequencing of 12 human exomes. Nature.

[btt003-B7] Sherry ST (2001). dbSNP: the NCBI database of genetic variation. Nucleic Acids Res..

[btt003-B8] Szklarczyk D (2011). The STRING database in 2011: functional interaction networks of proteins, globally integrated and scored. Nucleic Acids Res..

[btt003-B9] Tong H (2008). Random walk with restart: fast solutions and applications. Knowl. Inf. Syst..

[btt003-B10] Tyner JW (2009). RNAi screen for rapid therapeutic target identification in leukemia patients. Proc. Natl Acad. Sci. USA.

[btt003-B11] Tyner JW (2013). Kinase pathway dependence in primary human leukemias determined by rapid inhibitor screening. Cancer Res..

[btt003-B13] The 1000 Genomes Project Consortium (2010). A map of human genome variation from population-scale sequencing. Nature.

