# Supplementary material for: HitWalker: variant prioritization for personalized functional cancer genomics
Source: Bioinformatics. 2013 Jan 9;29(4):509–10. doi: 10.1093/bioinformatics/btt003 (PMC3570211; doi:10.1093/bioinformatics/btt003)
Supplement: Supplementary Data [file supp_29_4_509__index.html]

HitWalker: variant prioritization for personalized functional cancer genomics — HitWalker: variant prioritization for personalized functional cancer genomics — Supplementary Data 

# HitWalker: variant prioritization for personalized functional cancer genomics

## Supplementary Data

files

**Files in this Data Supplement:**

- Supplementary Data - doc file
- Supplementary Data - png file
