# Supplementary material for: HitWalker: variant prioritization for personalized functional cancer genomics
Source: Bioinformatics. 2013 Jan 9;29(4):509–10. doi: 10.1093/bioinformatics/btt003 (PMC3570211; doi:10.1093/bioinformatics/btt003)
Supplement: Supplementary Data [file supp_btt003_bioinf_app_note_supplementary_REV1_FINAL.doc]

**Rationale for random walk with restarts algorithm**

The utility of the random walk with restarts (RWR) algorithm for the problem of ranking sets of variants relative to functional assay results can be seen by first considering a simple approach. For a given protein-protein interaction network (PPI), we can first determine the set of proteins impacted by the variants and determine if the direct neighbors are members of the set of proteins with assigned functional assay scores (hit proteins). Variant proteins could then be prioritized based on some function of the confidence of the interactions, number of adjacent hit proteins and their score. This approach, while relatively simple to interpret, would suffer from a few issues. First, it would fail in the case of no direct interactions between variant proteins and hit proteins. Although this may not be an issue for a dense PPI database such as STRING (Szklarczyk *et al.* 2011), alternative manually curated databases could provide fewer interactions between proteins. Second, indirect paths would not be considered even if they were of higher confidence. Finally, there can potentially be many hit proteins with a score and this method would not consider rankings based on association relative to the entire set.

The RWR framework provides a mechanism with which to address these issues and is tractable even for large networks. It can be described in a biological context as follows. For a given PPI, values representing the importance of a given protein in the network are assigned first. For our context of functional assays these are the scores assigned to each protein based on the results of the functional assays. How to assign these scores is considered below. This set of proteins and scores, after being scaled to sum to one, represents the probability of (re)starting the random walk at that protein. So higher scores for a given protein would likely result in higher RWR association scores for adjacent proteins. The other main element of the RWR algorithm is the presence of scores indicating the level of evidence for the interaction between two proteins. Typically these scores are scaled by a function of the overall score totals for one or both proteins. In practice the manner in which the scaling is performed does change the resulting prioritization. Using the iterative algorithm outlined below, all proteins in a network have an association score attributed to them regardless of how distant they are from the nearest hit protein. This score intuitively is a function of both proximity to the hit protein and the confidence of the path from variant protein to the set of hit proteins. For these reasons, we believe variants of the RWR algorithm to be well suited to the problem or prioritizing variants relative to functional assay results.

**Details on the HitWalker Prioritization Algorithm**

Input to the random walk with restarts algorithm consists of several types of data. First a user specifies an association matrix *A* that denotes the association between members of the set of proteins, *R*, in a protein-protein network (PPI). Here an interaction between protein *i* and *j* would be set to the defined evidence score for the interaction or otherwise set to zero. In the simplest case the matrix would consist solely of ones and zeros indicating interaction or non-interaction respectively. All entries of the matrix should be between 0 and 1. From *A,* we define the matrix *P* that consists of the scaled interaction scores. We use a network propagation parameterization by default (Vanunu *et al.*, 2010), which is the matrix *P* is defined for each *i* adjacent to *j*:

(1)

Next, the user specifies a vector α that contains a score for each member of *R.* The scores are obtained from functional assays (or other measurements of interest) and typically will only be defined for a subset of *R* with the remainder set to zero. The elements of α are then scaled so that they sum to 1. A vector *χ* is defined which contains the computed RWR association scores for each at time *t.* This vector is set equivalent to α at *t*=0 and is then updated as follows:

(2)

Where the variable *r* denotes the probability of restart and is a user-defined value. Pragmatically, *r* is a measure of the importance placed on the functional assay scores and can influence the rankings. The vector *χ* is updated using (2) until convergence or until a specified number of iterations are reached. Finally, the user specified set of proteins containing variants is ranked using their respective values in *χ.* The RWR implementation is based off of publically available code (Erten *et al.* 2011). For implementation-specific details see the functions ‘random.walk’ and ‘run.protocol’.

**Defining inputs to RWR algorithm**

One of the fundamental aspects of HitWalker is how to assign scores to genes based on the results of functional assays. For our work, there are two main types of assays. One is based off of the use of siRNA to downregulate a predefined set of genes thought to be important for cancer cell viability (Tyner *et al.* 2009). We currently define hits as outliers relative to previous assay runs using a Z score cutoff with a function of cell viability as the outcome measure. If only this type of data exists for a given patient sample, we typically would use a binary encoding of hit proteins for α (i.e. 1 for hit, 0 for non-hit). However, this assumes that all of the siRNA genes should have equal weight in the RWR algorithm. An alternate way would be use a more quantitative encoding of these values such as the Z score for those reaching hit status. The other type of assay we commonly use involves treatment of cancer cells with a panel of drugs to determine sensitivity or resistance. Converting the resulting IC50 values to scores attributed to genes has been described previously (Tyner *et al.* 2012) and essentially takes into account specificity of drug targets and drug response. We typically use the drug scores directly so that initially the corresponding elements of α can take any positive real number. When a patient sample contains both siRNA hits as well as drug target scores we typically set all of the genes with siRNA hits equal to the maximum drug target score. Note that both the siRNA as well as the drug targets tend to be denoted as genes as opposed to transcripts or proteins and so a mapping between gene to protein symbols has to be performed. Each protein that corresponds to a gene is set to the raw score for that gene in the α vector prior to scaling.


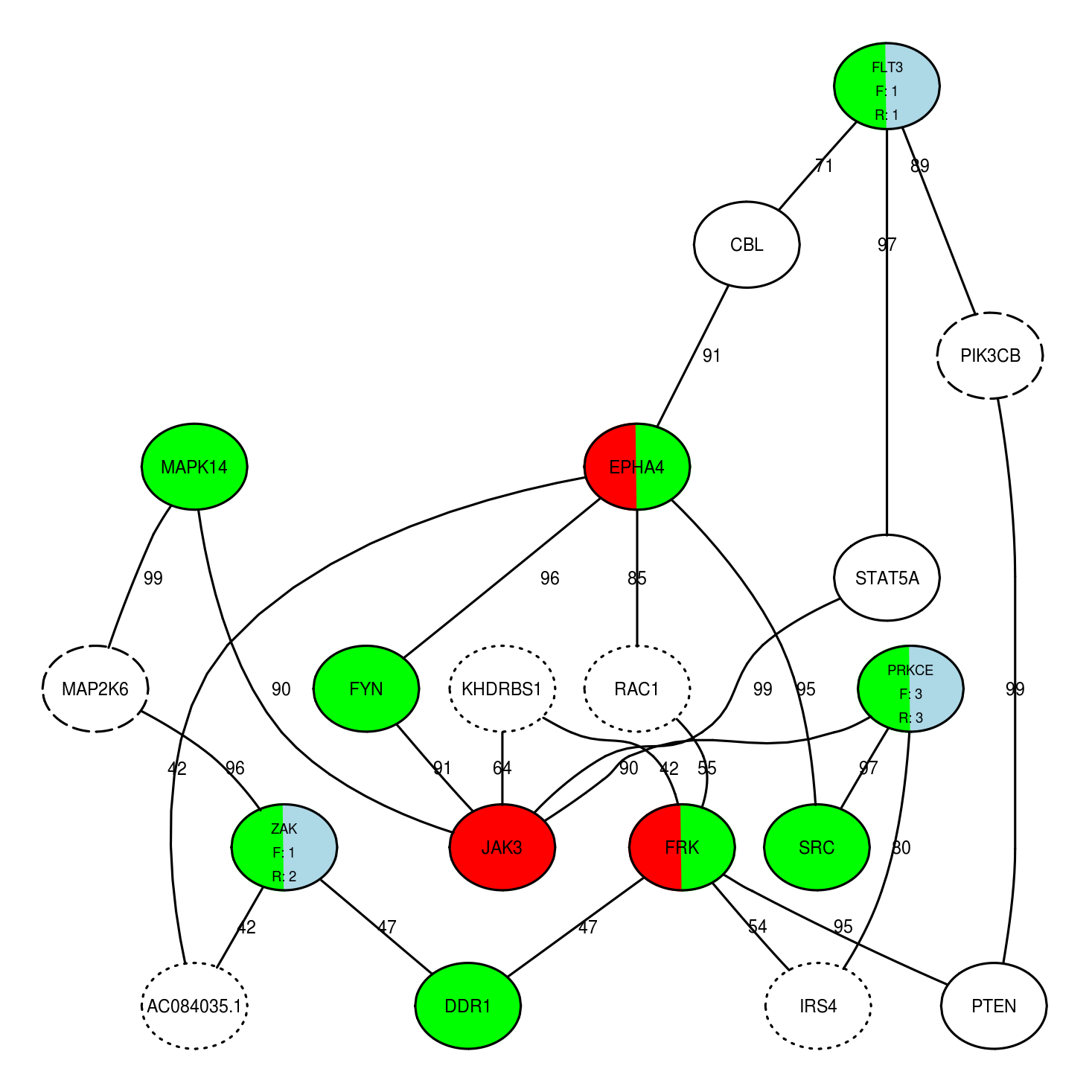


**Figure S1.**  Full visualization output from HitWalker displaying the top 3 assay hits (EPHA4, JAK3 and FRK) and variants (FLT3, ZAK and PRKCE) for an acute myeloid leukemia patient. Note that other hits are pulled out and annotated as they are on the shortest path. Gene names are provided for each node. For nodes containing variants (blue), frequency information is reported in terms of the patient cohort counts (F) as well as the RWR rank (R). Red and green nodes indicate siRNA and gene target hits respectively. Dotted borders indicate absence of capture probes for a given gene. Dashed borders indicate functional assay targets whose inhibition did not significantly alter cell viability. Confidence scores for the interactions between the two genes are reported near the lines connecting two given genes.

**References**

Erten,S., Bebek,G., et al. (2011) DADA: Degree-Aware Algorithms for Network-Based Disease Gene Prioritization. *BioData Mining*, 4, 19.

Szklarczyk,D., Franceschini,A., et al. (2011) The STRING database in 2011: functional interaction networks of proteins, globally integrated and scored. *Nucleic Acids Res.*, 39, D561-8.

Tyner,J.W., Deininger,M.W., et al. (2009) RNAi screen for rapid therapeutic target identification in leukemia patients. *Proceedings of the National Academy of Sciences*, 106, 8695-8700.

Tyner,J.W., Yang,W.F., et al. (2012) Kinase Pathway Dependence in Primary Human Leukemias Determined by Rapid Inhibitor Screening. *Cancer Res.*
