# Supplementary figures and images for: HitWalker: variant prioritization for personalized functional cancer genomics
Source: Bioinformatics. 2013 Jan 9;29(4):509–10. doi: 10.1093/bioinformatics/btt003 (PMC3570211; doi:10.1093/bioinformatics/btt003)

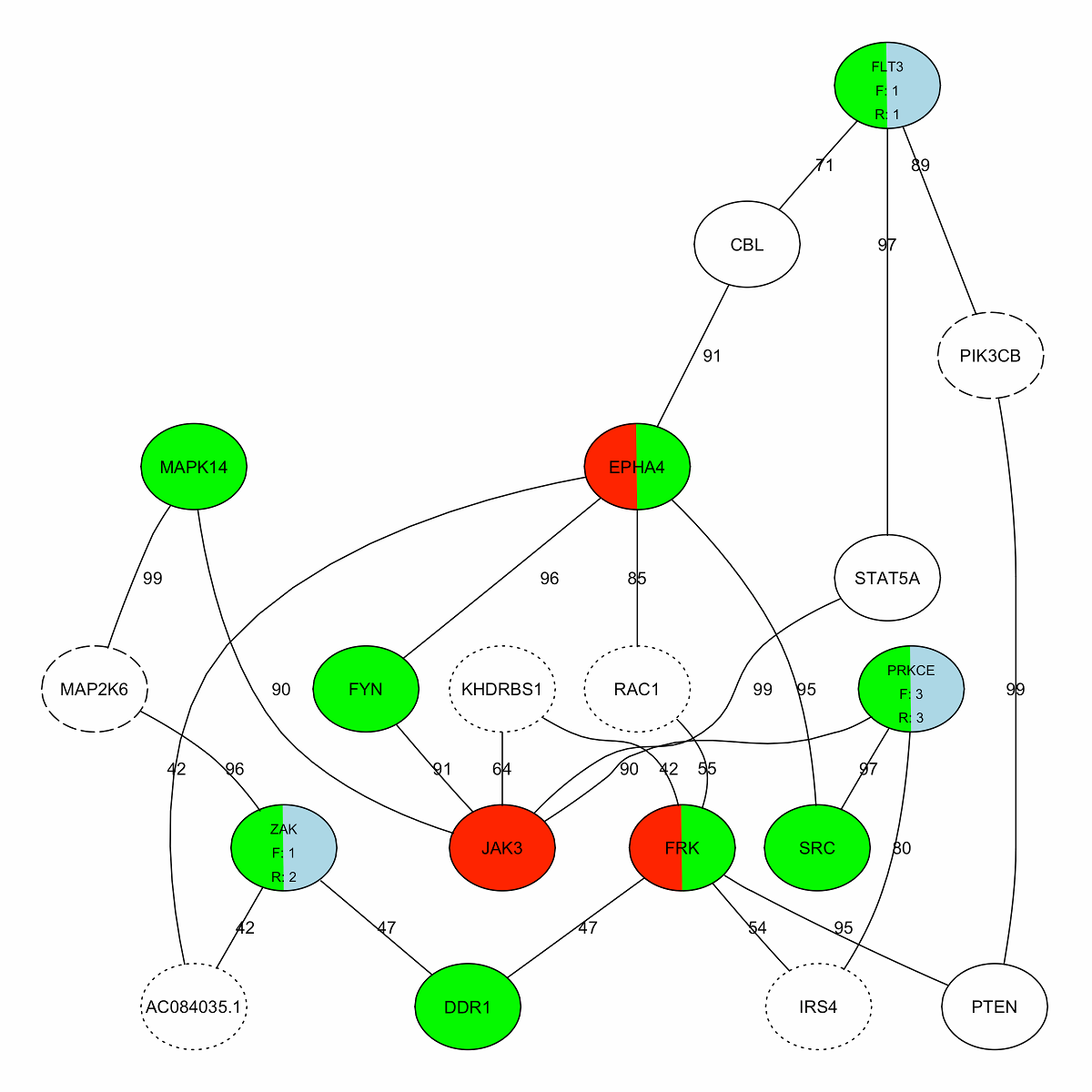

Supplement: Supplementary Data [file supp_btt003_FigureS1.png]
